# Broadly Reactive Real-Time RT-PCR Assay for the Detection of Hepatitis E Virus and Simultaneous Genotyping by Single Nucleotide Polymorphism Analysis

**DOI:** 10.1128/spectrum.01912-21

**Published:** 2022-02-09

**Authors:** Setsuko Ishida, Shima Yoshizumi, Hidekatsu Sakata, Keiji Matsubayashi

**Affiliations:** a Hokkaido Institute of Public Health, Sapporo, Japan; b Japanese Red Cross Hokkaido Block Blood Center, Sapporo, Japan; c Central Blood Institute, Blood Service Headquarters, Japanese Red Cross Society, Tokyo, Japan; Center for Research and Advanced Studies (CINVESTAV-IPN)

**Keywords:** HEV, hepatitis, genotype, hepatitis E virus

## Abstract

Hepatitis E virus (HEV) infection is a global public health concern. Although HEV infection is usually asymptomatic and self-limiting, extrahepatic manifestations and chronic infections in immunocompromised patients have been described. HEV strains infecting humans have been classified into four main genotypes. In this study we have developed and validated a novel sensitive real-time RT-PCR assay for the detection of all four HEV genotypes. Simultaneous discrimination of genotypes 1, 2, and 4 from genotype 3 by single nucleotide polymorphism (SNP) analysis was possible. In all, 201 serum samples from cases and carriers previously tested for HEV by nested RT-PCR were analyzed. Twenty-seven HEV-positive samples could not be typed by the nested RT-PCR and nucleotide sequencing, but were newly typed by SNP analysis. As polymorphisms were present at the primer or probe binding site, we adopted a degenerate primer and mixed probes. When a mixed probe was added, the fluorescence intensity increased, facilitating genotype determination.

**IMPORTANCE** The distribution of HEV-3 and HEV-4 has been changing. HEV-4, which had been predominantly found in Asia, is now being detected in other parts of the world, and there are now reports of chronic infections. Additionally, neurological disorders have frequently been reported in patients with acute or chronic HEV infections. HEV-4 has also been shown to lead to a higher severity in terms of acute hepatitis than does HEV-3. Early typing can provide useful information regarding the route of infection and for tailoring treatment to the expected course of the disease. The present method afforded a good detection rate even when polymorphisms were present within the target region for viral gene detection. We believe that this method can be applied to the analysis of mutation-prone viral genes in the future.

## INTRODUCTION

Hepatitis E virus (HEV) is the most common cause of acute viral hepatitis around the world. This virus infects estimated 20 million people every year and was a cause of more than 3.3 million acute hepatitis E cases, and over 44,000 hepatitis E-related deaths in 2015, mainly in developing countries (1). HEV infection is typically asymptomatic and self-limiting, but chronic infections in immunocompromised patients have been reported (2). Additionally, HEV infection has been associated with extrahepatic manifestations (2). Among the eight genotypes, HEV strains infecting humans have been classified into four main genotypes belonging to a single serotype (3, 4). Genotypes 1 and 2 are endemic in Africa and Asia. These are transmitted between humans by the fecal-oral route, and are responsible for large waterborne outbreaks in developing countries. Genotypes 3 and 4 have been reported to cause zoonotic infections in humans from infected pigs, boars, and deer (5). Transmission usually occurs through the consumption of raw or inadequately cooked pork. The potential for zoonotic transmission of rabbit HEV (HEV-3ra) (6), boar HEV (HEV-5) (7), camel HEV (HEV-7) (8) those belonging to *Orthohepevirus A* (HEV-A), and another species of rat HEV (*Orthohepevirus C*, HEV-C) (9, 10) has also been reported, although in limited numbers. Whether boar HEV (HEV-6) and camel HEV (HEV-8) are transmissible to humans remains unclear. Transmission of HEV via blood transfusion and transplantation has also been documented (11–15).

When a phylogenetic tree is drawn with human HEV strains identified from the same geographic region, most of them tend to cluster together, but a few strains from the same geographic region can differ significantly in terms of their genomic sequence (3, 16–18). In order to understand the distribution of HEV variants, it is necessary to identify the HEV subtype. HEV-3 was first reported as a human case in the U.S. (3) and, thereafter, identified almost worldwide. The distribution of its 10 subtypes (HEV-3a to HEV-3j) varies widely. HEV-3a and HEV-3j strains circulate in North America and Australia; HEV-3b, HEV-3d, and HEV-3g strains are found in Asia; and HEV-3c, 3e, 3f, 3h, and 3i circulate in Europe (19–24). In Japan, HEV-3a, 3b, and 3e strains circulate in humans and pigs (25, 26). Nine HEV-4 subtypes (HEV-4a to HEV-4i) have been mostly isolated in Southeast Asia and China. Among them, HEV-4c and 4b are predominant in Japan (25, 26). HEV-4 strains were frequently detected in Hokkaido, Japan, and detection includes cases of transfusion-transmission (11, 12, 16).

HEV-3 is the most extensively studied genotype with regard to chronic infection. It is associated with chronic liver disease and cirrhosis in immunosuppressed individuals, such as solid organ transplant recipients, human immunodeficiency virus (HIV) infected patients, and those with hematological diseases (27, 28). Additionally, neurological disorders have frequently been reported in patients with acute or chronic HEV infections (29). Recently, chronic HEV infections caused by HEV-4 have been reported in a female liver transplant recipient from Taiwan (30), and in a patient from the United States with a fatally accelerated cirrhosis (31). It was reported that the severity of illness in cases with HEV-4 infection is greater than that in those with HEV-3 infection in Japan and France (32–34). Further studies are needed to determine if differences in the hepatitis E presentation and outcome might be linked to the HEV genotypic patterns. If HEV-3 is more likely to establish chronic infection and HEV-4 is more likely to result in severe illness, early discrimination of HEV-3 and HEV-4 might be useful in tailoring treatment to the expected course of the disease. In addition, in developed countries, most hepatitis E is caused by zoonotic genotype 3. If a genotype that does not originally exist in the area is detected, it is assumed that it was brought in from outside the region through travel. Such information is useful for estimating the route of infection.

The detection of RNA in clinical samples by reverse transcriptase (RT)-PCR allows both diagnosis and genotyping. For reliable diagnosis, a combination of antibody detection and nucleic acid-based assays has been recommended (35, 36). Nested RT-PCRs followed by sequencing can determine the HEV genotype and subtype, which will help to identify the source of infection, trace the route of transmission and interpret the epidemiology. Considering the heterogeneity of the HEV strains detected in humans and animal species, and to monitor HEV RNA in various types of samples such as serum, feces, and environmental samples, several conventional RT-PCR and real-time RT-PCR assays have been developed (37–41). Currently, a real-time RT-PCR assay, developed in 2006 (40), is the most widely used for the detection of HEV infection in humans (42, 43), and this assay is capable of detecting all four genotypes. The assay targets the ORF2-ORF3 overlap region and is designed for sensitive and rapid detection of the zoonotic HEV genotypes. This assay aid epidemiological investigations and enable us to understand the situations of outbreaks.

We have developed a novel sensitive real-time RT-PCR assay for all four HEV genotypes from various samples. In this method, HEV genotypes are discriminated simultaneously by single nucleotide polymorphism (SNP) analysis. We have successfully validated this method against a panel of defined clinical samples and swine liver samples containing several subtypes of HEV. This assay was found to be able to discriminate between HEV-3 and HEV-4 in clinical samples, and the results were consistent with those obtained by nucleotide sequencing. This novel assay is predicted to be helpful in the investigation of suspected HEV infections, and provide a powerful tool for epidemiological investigations and the evaluation of risks associated with the consumption of pork products.

## RESULTS

### Validation of SNP analysis.

All results of the SNP analysis were compared with those obtained from the conventional nested RT-PCR. Amplification of the ORF1 and ORF2 regions was performed for 201 serum samples from cases and carriers for HEV by nested RT-PCR previously. Then, the HEV genotype was determined by sequencing of the ORF2 region. If only the ORF1 region was amplified, the sample was also considered HEV positive. However, the amplified product of the ORF1 region is 104 to 118 bp, which is shorter than that of the ORF2 region (420 bp), so it is not suitable for genotyping by sequencing. Overall, 24 samples were regarded as testing positive based on the amplification of the ORF1 region by nested RT-PCR, but the genotype was not determined for these samples (Table 1).

**TABLE 1:** Comparison of the performance of real-time RT-PCR assays and the nested RT-PCR in the detection of HEV in clinical samples

Assay	No. of samples negative	No. of samples positive only by ORF1 primers	No. of samples positive for HEV-3	No. of samples positive for HEV-4
Nested RT-PCR	35	24	74	68
Real-time RT-PCR	33		82	86

Sixty-eight serum samples tested positive for HEV-4 by both nested RT-PCR and SNP analysis (Table 1), and 74 serum samples tested positive for HEV-3 by both nested RT-PCR and SNP analysis. Thus, the newly developed SNP analysis confirmed the results obtained by nested RT-PCR and sequence analysis. Altogether, discrepancies among results were observed for 29 samples. First, the SNP analysis was able to distinguish genotypes in 23 of the 24 samples that could not be typed on the amplification of the ORF1 region by nested RT-PCR and sequencing (Table 1). For instance, eight samples were newly positive for HEV-3, bringing the number of HEV-3-positive samples from 74 to 82. A further 15 were newly positive for HEV-4, bringing the number of HEV-4-positive samples from 68 positive to 83. One sample only amplified by ORF1 primers turned out to be negative. Among the 35 samples testing negative by nested RT-PCR, one was genotyped as HEV-3 by the SNP analysis, bringing the number of HEV-3-positive samples to 83, and three were genotyped as HEV-4 bringing the total number of HEV-4-positive samples to 86. Finally, 27 HEV-positive samples could not be typed by the nested RT-PCR and nucleotide sequencing, but were newly typed by SNP analysis. One sample was positive for HEV-3 by nested RT-PCR but tested negative by SNP analysis, bringing the total number of HEV-3-positive samples to 82. The results of genotyping by nucleotide sequence following nested RT-PCR for swine liver samples and those of real-time RT-PCR were also in agreement.

**FIG 1:**
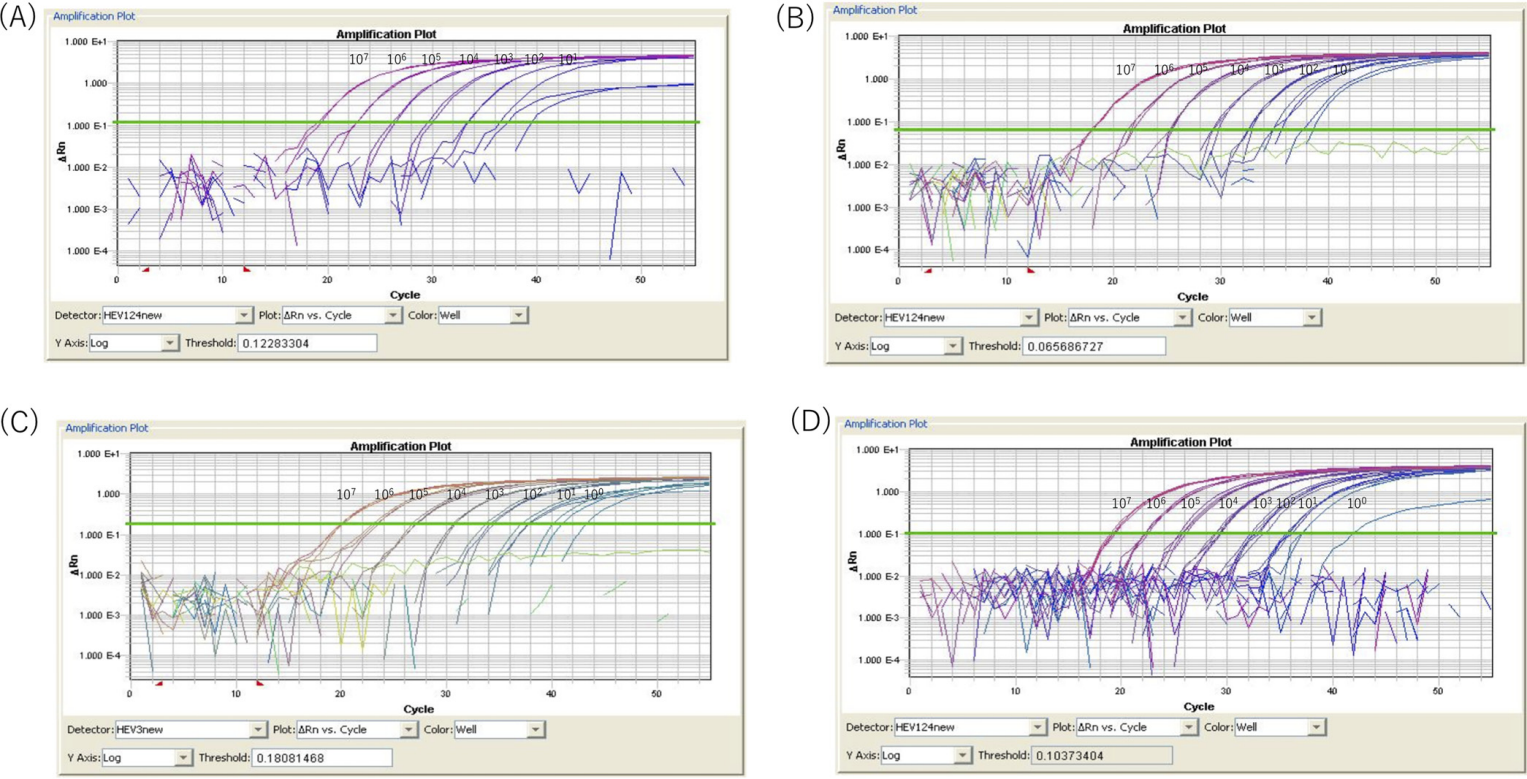
Testing of serial dilutions (1 × 10^7^ to 1 × 10^0^ copies/reaction tube) of control plasmids with inserts of the HEV sequences by real-time RT-PCR. Control plasmids with the HEV-1, 2, and 4 sequences reacted with the HEVP124VIC and HEVP124VICk probes (A, B, and D). Control plasmids with the HEV-3 sequences reacted with the HEVP3-2FAM probe (C).

Using positive controls serially diluted from 1 × 10^7^ to 1 × 10^1^ copies in the SNP analysis, genotypes 1 to 4 were correctly identified as positive for HEV. The fluorescence curves for the genotypes 1 to 4 DNA controls shown in Fig. 1 demonstrate that this assay is efficient, in semi-quantitative terms, at amplifying the HEV templates.

### Genotyping by SNP analysis.

**FIG 2:**
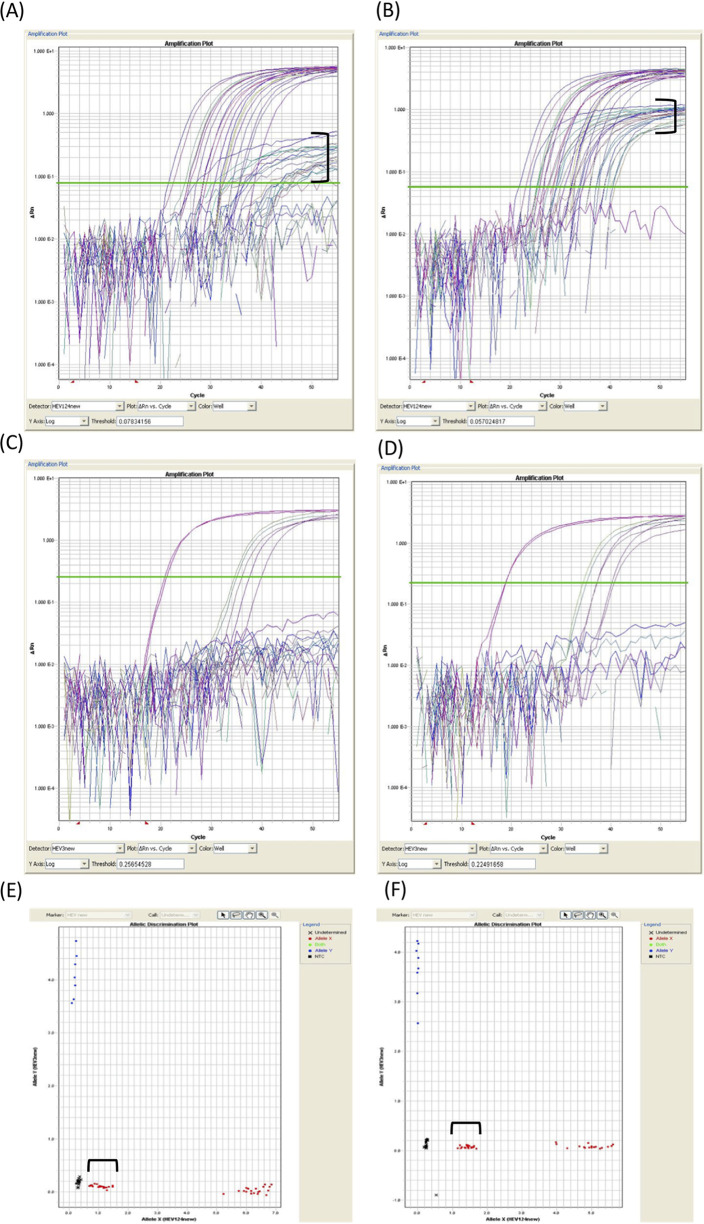
Genotyping of HEV by SNP analysis of 65 clinical samples, including samples with HEV-4c polymorphisms. The amplification curves and scatterplots obtained using the mixed probe (HEVP124VIC and HEVP124VICk) are shown on the right (B, D, and F), and the amplification curves and scatterplots obtained using only HEVP124VIC are shown on the left (A, C, and E). VIC fluorescence was detected by the HEV-1, 2, and 4 sequences (A, B), and FAM fluorescence was detected by the HEV-3 sequence (C, D). The amplification curve in the square brackets shows that the fluorescence intensity was enhanced by using the mixed probe (B). This improved the separation of the samples in the square brackets from the negative control in the scatterplot (F).

**FIG 3:**
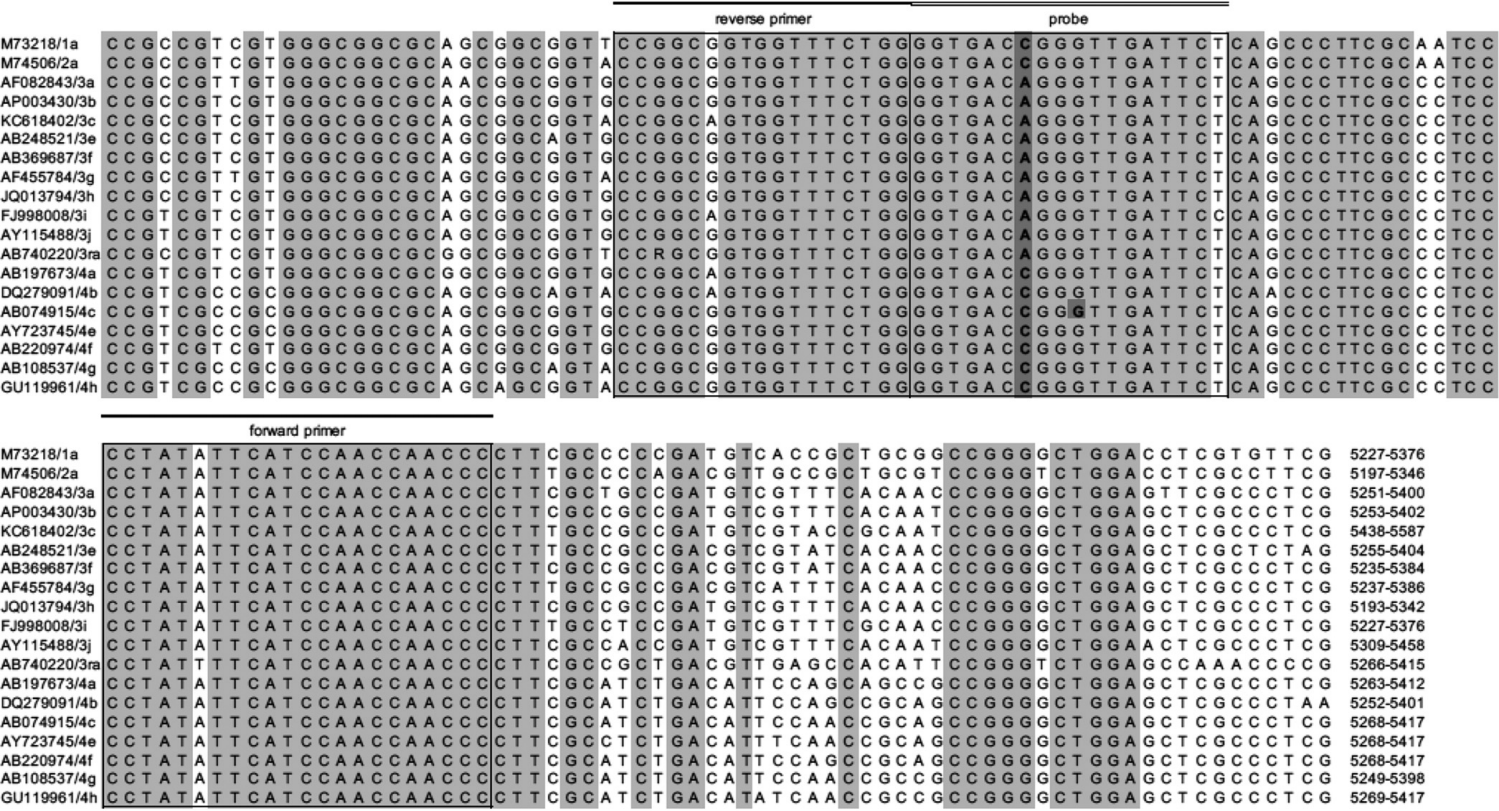
Alignment of HEV strain sequences used to design the primers and probes. The sequence position corresponds to 5261 to 5330 based on GenBank accession no. M73218. The SNP sites that distinguish HEV-1, 2, and 4 from HEV-3 and the polymorphism found in HEV-4c are shaded.

The amplification results of the real-time RT-PCR were plotted on a scatterplot of HEV-1, 2, and 4 (*x* axis) versus HEV-3 (*y* axis) using the Allelic Discrimination software (Fig. 2). Evaluation using the newly developed SNP analysis revealed that some of the HEV-4 strains showed low fluorescence intensity. Nucleotide sequence analysis and BLAST search showed a common polymorphism of G to A at the probe-binding site of HEV-4c strains (Fig. 3). Therefore, a new probe, HEVP124VICk, was designed and the mixing proportions of HEVP124VIC and HEVP124VICk were examined. The ratio of 4:1 was chosen as the fluorescence intensity was increased in the strains showing the polymorphism, making it easier to distinguish them from the negative control (Fig. 2, [A] versus [B], [E] versus [F]).

## DISCUSSION

In this study, a novel method of SNP analysis for discriminating between HEV-1, 2, and 4 and HEV-3 was developed and validated using archived serum and liver samples. The sensitivity of the semi-quantitative real-time RT-PCR assay was comparable with or better than that of conventional nested RT-PCR assay (Table 1). The nested RT-PCR method using the ORF1 primer is sensitive in detecting viral genes, but the amplified product was of insufficient length for genotyping by sequencing. The SNP analysis results were compared with those of nucleotide sequencing, and no discrepancies in genotyping were found between the assays. The real-time RT-PCR assay developed in this study could be performed rapidly and with appropriate sensitivity, suggesting that this assay affords a powerful diagnostic tool.

A few discrepant results between samples found to be positive by nested RT-PCR method but negative by SNP analysis were observed, and it is thought that such discrepancies occurred due to the virus load being close to the detection limit. Another possibility is that the negative results were related to the degradation of the nucleic acids by several freeze/thawing cycles during storage.

One limitation of this study is that we were not able to validate any samples other than those stored. However, alignment of the primer/probe regions of the nearly complete/complete sequences of 575 strains as reference sequences for HEV-1/2/3/4 (24) suggested that our method could detect and type most of the strains (data not shown). When using our method, if the fluorescence intensity is low due to some mismatches, confirmation by nested RT-PCR and sequencing is necessary.

Multiple sequence alignment showed that the highest nucleotide homology was found in the ORF2-ORF3 overlapping region, and various HEV primer/probe sets have been set up in this region for the detection and quantification of HEV RNA (44, 45). However, novel single nucleotide polymorphisms could also be present within the primer and probe binding sites. This raises a potential risk of failed detection or under-quantification of HEV RNA levels. In fact, recent studies has identified polymorphisms in the probe-binding site of the most widely used real-time RT-PCR assay for HEV detection (42, 46): a C-to-T single nucleotide mutation in the probe-binding site of this assay (40) was reported to be associated with the risk of false-negative RT-qPCR results (47). As reported by other researchers, degenerate primers may be less sensitive than non-degenerate primers for the following reasons. This is because the concentration of individual primer is lower, and it is more difficult to estimate the consensus Tm of the degenerate primers (48, 49). Essentially, SNP analysis is strictly designed to control the Tm values of the two primers and the two probes. The use of a degenerate primer and the mixing of the probes was a difficult challenge, but confirmation of an efficient probe mixing ratio facilitated the discrimination of negative and positive results in this study. The primer sequences should be checked by comparison the epidemic strains and improved as needed. Viruses are highly mutable, but we believe that such a methodology could afford a breakthrough in virus analysis.

This polymorphism may be related to the evolutionary divergence between HEV-3 and the other genotypes, HEV-1, 2, and 4. The sequence of this primer/probe region is one of the most highly conserved across the whole HEV genome. The SNP site in the probe-binding site, which is the basis for typing HEV-3 and HEV-1, 2, and 4 in this study is A in HEV-3 and HEV-7 (KJ496144), and C in HEV-1, HEV-2, HEV-4, HEV-5 (AB573435), HEV-6 (AB856243), and HEV-8 (KX387867). This is consistent with divergence in the molecular phylogenetic tree drawn from the whole genome sequence of HEV (50). It seems contradictory that most of these nucleotide changes represent silent mutations and do not result in significant differences at the amino acid level. In fact, the SNP sites within the probe-binding site in this study encode the same amino acid, Arg. However, the polymorphic site in the HEV124VICk probe encodes Val in most HEV-3 and HEV-4 strains, Ile in the HEV-4c mutant strain and Ala in a pig strain. These amino acids are small and share the same polarity, so the nature of the protein may not differ greatly.

Although our method requires sequence analysis for the detailed tracing of the route of transmission, it is a powerful starting point for analyses associated with epidemiological studies. The number of reports of virus typing by SNP analysis has increased in recent years. We have previously reported a duplex TaqMan RT-PCR method for norovirus detection/genotyping (51). Additionally, SNP analysis has also been applied to the determination of drug resistance among influenza viruses (52–55).

Our goal was to develop a highly sensitive and specific assay that detects all four genotypes and can be used for direct genotype differentiation without sequencing. Serological assays are available for screening large numbers of human serum samples. On the other hand, real-time RT-PCR assays are more informative because they can be applied to each type of samples from a variety of animal species and environmental sources.

### Conclusions.

In conclusion, the new real-time RT-PCR assays provides a powerful tool to improve the diagnosis and investigation of HEV infections, an important foodborne and waterborne disease in humans. In addition, the assays are useful for the detecting of HEV in swine and game species that cause zoonotic infections, as well as in environmental samples. A method of detecting HEV with simultaneous genotyping is also of particularly useful in epidemiological studies. The use of degenerate primers and mixed probes is disadvantageous in SNP analysis where strict temperature control is required, but this disadvantage can be overcome. The techniques described here allow the detection all four genotypes and can be used for screening large numbers of samples. The information these assays can provide has the potential to strengthen control programs for HEV, an important zoonotic disease occurring worldwide. The high sensitivity, simplicity, and reproducibility also make these assays suitable for diagnostic use in routine laboratories.

## MATERIALS AND METHODS

### Experimental samples.

A panel of 201 serum samples, consisting of 166 samples previously testing positive and 35 samples testing negative for HEV by conventional nested RT-PCR, were selected for evaluation of the assay’s specificity. These samples were collected over an 11-year period (2004 to 2014) from sporadic cases of acute hepatitis or carriers identified by nucleic acid amplification screening for HEV among blood donors at the Japanese Red Cross Blood Center in Hokkaido (56). Viral RNA was extracted from serum samples by a spin column technique using the QIAamp MinElute Virus Spin Kit (Qiagen, Hilden, Germany) according to the manufacturer’s protocol. Also, four swine liver samples found to be positive, and for which the genotype was confirmed, were used in this study. Liver RNA was extracted from a piece of each specimen (3 mm × 3 mm × 3 mm) using a RNeasy mini kit (Qiagen) (16). The conventional nested RT-PCR was performed according to the previously described protocol with sets of primers targeting a highly conserved sequence within HEV open reading frame (ORF) 1 and ORF2. The primers used were: HE7-1/HE7-2/HE7-3/HE7-4, HE7-5/HE7-6/HE7-7/HE7-8/HE7-9 primers for ORF1 (57) and HE040/HE044 and HE041/HE110-2 primers for ORF2 (58). The genotype of the amplified products was classified according to the nucleotide sequence of ORF2. The gene subtypes of these serum samples included 3a, 3b, and 3e for HEV-3, and 4b, 4c, and 4f for HEV-4, while the gene subtypes of the swine liver samples were HEV-3b and HEV-4c.

### Primers and probes for the SNP analysis.

The primer and probe sequences are listed in Table 2. Primers and probes for the SNP analysis were designed based on a multiple sequence alignment of HEV genome sequences available in GenBank (Fig. 3). A pair of primers (F34-2 and R34-3m) and two probes (HEVP124VIC and HEVP3-2FAM) located in the conserved ORF2-ORF3 overlap region and broadly reactive with HEV genotypes 1 to 4 were selected. R34-3m is a degenerate reverse primer, as there is a polymorphism present in the region of primer-binding site. Two probes were designed to discriminate the C nucleotides of HEV-1, 2, and 4, and the A nucleotide of HEV-3 gene, respectively. A new probe, HEVP124VICk, was added to the evaluation as a polymorphism in the region of the probe-binding site of HEV-4c strains was found by sequence analysis in a certain percentage of the clinical samples (Fig. 3). By using HEV-4c strains showing polymorphisms, we were able to compare the amplification results without the HEVP124VICk and different mixing ratios of HEVP124VICk. The mixing ratio of HEVP124VIC and HEVP124VICk was changed to 2:1 and 4:1.

**TABLE 2:** Primers and probes used for real-time RT-PCR

Primers and probes	Sequence (5′–3′)
Forward primer HEVF34-2	5′-GGGTTGGTTGGATGAATATAGG-3′
Reverse primer HEVR34-3m	5′-CCGGCRGTGGTTTCTGG-3′
Genotype 3 probe HEVP3-2FAM	5′-FAM-AGAATCAACCCTGTCAC-MGB-3′
Genotype 124 probe HEVP124VIC	5′-VIC-AATCAACCCGGTCAC-MGB-3′
Genotype 124 probe2 HEVP124VICk	5′-VIC-ATCAATCCGGTCACC-MGB-3′

### Positive control and sensitivity analysis.

Four positive control plasmids were constructed by amplifying a genomic region corresponding to nucleotide positions 5257 to 5427 of a HEV-1 human HEV strain (M73218) from clinical samples of the HEV-1, HEV-2, HEV-3, and HEV-4 strain, respectively. The sensitivity of the assay was evaluated by generation of concentration response curves using 10-fold serial dilution of the four positive control plasmids. Each diluted sample was tested in at least triplicate and the serial dilution was repeated at least twice.

### Genotyping by SNP analysis following real-time RT-PCR.

Reverse transcription for the real-time RT-PCR assay was performed using the SuperScript III kit (Invitrogen). The reaction mixture (30 μL) contained RNA, first-strand buffer (50 mM Tris-HCl pH 8.3, 75 mM KCl, 3 mM MgCl_2_), 5 mM DTT, 50 ng random hexamers, 500 nM each deoxynucleoside triphosphate (dNTP), and 300 U SuperScript III reverse transcriptase. The mixtures were incubated at 25°C for 5 min, followed by 50°C for 60 to 90 min and 70°C for 15 min. The reaction time was varied in consideration of the patient’s symptoms, age, and the interval between the date of onset and the date of sample collection.

The real-time PCR was performed using a Type-it Fast SNP Probe PCR Kit (Qiagen) according to the manufacturer’s instructions. Briefly, the 25 μL assay contained 12.5 μL 2 × Master Mix, 2 μL Q solution, 2.25 μL of each of two 10 mM primers, 0.8 μL 5 mM reverse probe (HEVP124VIC), 0.2 μL 5 mM reverse probe (HEVP124VICk), 1 μL 5 mM forward probe (HEVP3-2FAM), and 4 μl cDNA. Thermal cycling was performed as follows: initial denaturation for 5 min at 95°C, followed by 55 cycles for amplification (denaturation at 95°C for 15 seconds and annealing as well as extension at 60°C for 45 seconds) using a 7900HT Fast real-time PCR system.

For SNP analysis, real-time RT-PCR data were collected after the reaction and analyzed to differentiate genotypes by the Allelic Discrimination software in the Sequence Detection System. Negative controls were included in each run.

### Ethics statement.

In accordance with the Law Concerning the Prevention of Infectious Diseases and Medical Care for Patients of Infections in Japan, hepatitis E is defined as mandatorily notifiable infectious disease, and samples from patients suspected of having hepatitis E can be collected and tested for HEV without informed consent from the patients. The Ethics Committee of Hokkaido Institute of Public Health approved the study on June 24, 2012. Blood donors were informed that samples might be used for epidemiological studies.
